# Doctor-diagnosed sleep disorders in the United States: Prevalence and impact of tobacco smoke exposure and vitamin D deficiency. A population-based study

**DOI:** 10.3389/frsle.2023.1113946

**Published:** 2023-04-03

**Authors:** Philip Kum-Nji, Samuel Taylor, Bah Tanwi

**Affiliations:** ^1^Children's Hospital of Richmond at the Virginia Commonwealth University School of Medicine, Richmond, VA, United States; ^2^Department of Neurology, Virginia Commonwealth University School of Medicine, Richmond, VA, United States; ^3^Specialist Neurologist in Private Practice, VMP, Prince George, VA, United States

**Keywords:** sleep disorders, risk factors, tobacco smoke exposure, cotinine, vitamin D

## Abstract

**Background and purpose:**

We determined the prevalence of physician-diagnosed sleep disorder and its association with tobacco smoke exposure and vitamin D deficiency.

**Methods:**

The National Health and Nutrition Examination Survey (NHANES) of 2011–2012 data base was used for the study. Subjects were asked two questions: “Ever told your doctor you had trouble sleeping?” and “Ever told by doctor have sleep disorder?” The answer “yes” to the second question indicated presence of a doctor-diagnosed sleep disorder (DSD) and “no” indicated its absence. Tobacco smoke exposure was defined by serum cotinine levels while vitamin D levels were measured by serum 25(OH) D. Eight selected variables included in the analyses were BMI, age, gender, smoking exposure, vitamin D levels, income, insurance, and race. Univariate and multivariate analyses were conducted to determine if tobacco smoke exposure and Vitamin D were each predictive of DSD.

**Results:**

Of 5,470 subjected aged 16 to 80+ years about 9% had doctor-diagnosed sleep disorder (DSD). In a multiple regression analysis, active tobacco smoking was predictive of DSD (OR 1.92; 95% CI = 1.38–2.69), while passive smoke exposure was not, even after controlling for all the other significant variables (OR 0.93; 95% CI = 0.57–1.52). The other variables significantly associated with DSD were by order of importance BMI (*P* < 0.001), Age (*P* < 0.001) and race (*P* ≤ 0.001). Vitamin D deficiency was not predictive of DSD.

**Conclusion:**

The prevalence of physician-diagnosed DSD was about 9%. Active smoking but not passive smoking as defined by cotinine levels was significantly associated with DSD. Vitamin D was not predictive of DSD. Future studies are therefore needed to demonstrate whether smoking cessation could help reduce DSD.

## Introduction

Sleep disorders are very common conditions in the United States and in the world. More than 30 years ago, Young et al. (1993) demonstrated the unexpected high prevalence of sleep disordered breathing among the US adult population with 9% occurring among women and up to 20% among men depending on the population studied and definitions used (Young et al., 1993, 2002).

One of the most common of these sleep disorders is obstructive sleep apnea (OSA) which typically manifests as the triad of snoring, witnessed breathing pauses and/or gasping/choking respirations during sleep and excessive daytime sleepiness (Durán et al., 2001). Other causes of sleep disorder are restless legs syndrome, various parasomnia of sleepwalking, sleep talking, groaning, nightmares etc. Other effects of sleep disorders on the wellbeing of individuals such as depression, anxiety, suicidal ideation, and even dementia have been well documented (Partinen and Guilleminault, 1990; Naqvi et al., 2014). Furthermore, sleep disorders are related to various cardiovascular morbidity and mortality (Shi et al., 2018). More recently, sleep disorders have even been shown to lower the threshold for seizures (Baker-Smith et al., 2021). Well known predictors of sleep disorders are obesity, age, sex, and race/ethnicity (Young et al., 1993; Jackson et al., 2013). Some smaller studies have recently demonstrated the role of vitamin D in sleep problems among adults (Stamatakis et al., 2007; Grandner et al., 2010; Sivathamboo et al., 2018).

Surprisingly, few recent national population-based studies in the US exist exploring the prevalence of sleep disorders and associated risk factors (Peppard et al., 2013). Furthermore, previous epidemiologic studies have relied mainly on self-reported information of sleep disturbances without objective evaluation by the physician for accuracy (Stamatakis et al., 2007; Grandner et al., 2010; Sivathamboo et al., 2018). The present population-based study, therefore, was aimed at determining the prevalence and risk factors for DSD in a large representative sample of adults living in the United States and the District of Columbia (DC). In particular, the role of tobacco smoke exposure and vitamin D status are two risk factors for DSD that have not been adequately explored in large population-based studies. Also, large population-based studies determining tobacco smoke exposure have relied mainly on self-reports of tobacco smoke exposure without validation by biomarkers such as hair, serum, or urine cotinine levels.

## Methods

### Ethics statement

The 2011–2012 NHANES Data set previously de-identified by the CDC was downloaded from the CDC website and used for our analyses. The VCU IRB approved the study as expedited.

### Design and study population

The 2011–2012 CDC National Health and Nutrition Examination Survey (NHANES) database was used for this study. The NHANES is a population-based comprehensive health assessment of US citizens in all 50 states including the District of Columbia. It is an ongoing two-yearly health assessment that includes survey data including physical examination, and laboratory evaluation of a nationwide representative sample of the US population. Some minority groups are oversampled by the cluster sampling techniques in order to obtain more accurate information of previously unstudied variables in these population subgroups.

During the interview survey, socio-demographic information was obtained on all household members sampled. Furthermore, blood samples were collected from these subjects in the mobile examination centers (MEC) in order to measure serum cotinine levels and vitamin D levels. Serum cotinine levels were measured using the isotope dilution-high performance liquid chromatography as described by Watts et al. (1990). The NHANES data set of 2011–2012 was used in the analyses. Data emerged included socio-demographic information, serum cotinine levels, vitamin D levels and sleep-disordered breathing.

**Figure 1:**
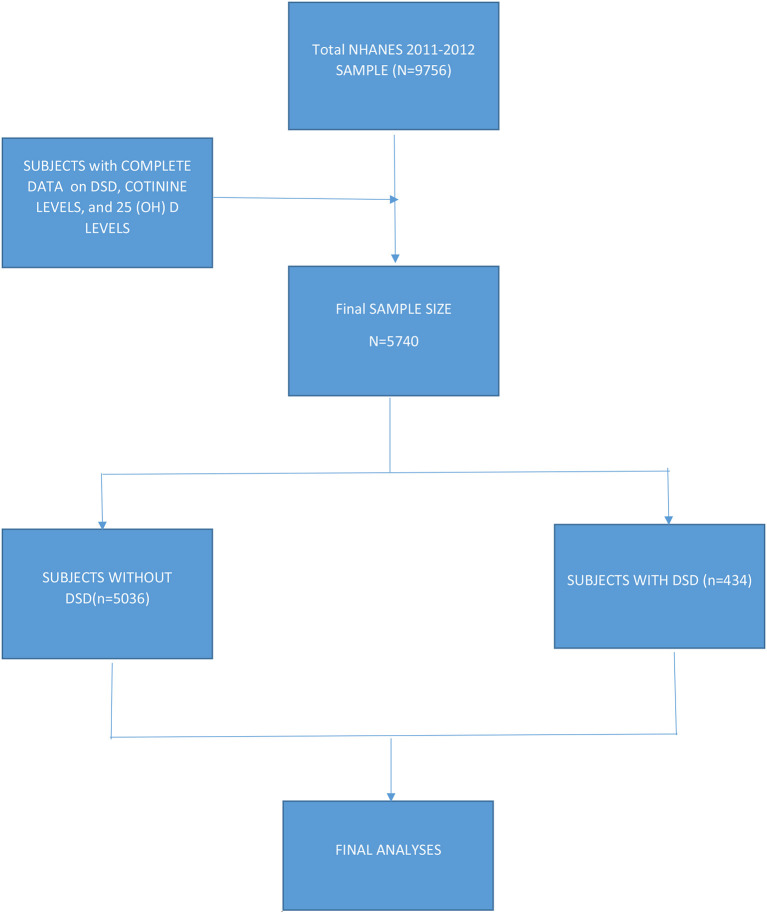
Flow chart of subject selection.

Data included subjects from all 50 states including the District of Columbia. Subjects were asked if they had ever told their doctor that they had sleep problems and they were further asked whether their doctor had diagnosed them with a sleep disorder. Of the 9,756 subjects in the 2011–2012 database, data on DSD, cotinine levels, and vitamin D (25(OH)D) levels were available for 5,470 subjects 16 years + (also see Figure 1).

### Measures and definition of terms

#### Tobacco smoke exposure

Tobacco smoke exposure was classified into three levels: subjects unexposed to smoking (cotinine levels < 0.05 ng/mL), subjects only exposed to smoking i.e., second hand smoke (SHS: cotinine levels 0.05 < 10 ng/mL), and subjects who were active smokers (cotinine levels = >10 ng/mL) as previously determined (Watts et al., 1990; Benowitz et al., 2009).

#### Body mass index

BMI for each adult subject including (those 16–18 years) was determined by the standard calculations of weight in kg/height in meters squared. BMI was further categorized into 5 groups: underweight and/or normal with BMI < 25; overweight were those with BMI 25–29.9; class 1 obesity were those with BMI of 30–34.9; class 2 obesity were those with BMI of 35–39.9; and class 3 were those with BMI in 40+ range.

#### Doctor-diagnosed sleep disorder

Subjects were asked two questions: (1) “Ever told your doctor you had trouble sleeping?” and, (2) “Ever told by doctor have sleep disorder?” Those answering “yes” to the second question were considered to have DSD while those who answered “no” were negative for DSD regardless of whether they had complained about sleep issues.

#### Vitamin D levels

Vitamin D (25(OH)D) levels were determined by ultra-high-performance liquid chromatography-tandem mass spectrometry. Details of the 25(OH)D assay methodology have been described elsewhere (Pirkle et al., 1996). Vitamin D deficiency was categorized as 25(OH)D of < 20 (deficient); 20–29.99 (insufficient); normal (sufficient) vitamin D 25(OH)D of ≥30 ng/mL (Yetley et al., 2010).

#### Age

For the purpose of this study, adult age in years was empirically categorized as follows: teen years (< 20 years); young adult (20–35 years); middle age (36–54 years); and old age (55+years). For a graphic presentation, we further categorized age by 5-year intervals in order to gain insight as to how age impacted DSD.

#### Other demographic data

The other pertinent sociodemographic variables previously studied with respect to DSD included age, annual family income, race/ethnicity, gender, and insurance status.

### Statistical analyses

As previously stated in the Methods section, the NHANES uses stratified cluster sampling techniques in its data collection procedure. Therefore, to obtain unbiased estimates representative of the US population, the present analyses were done using the complex sample analysis software of the IBM SPSS Statistics for Windows version 27, Armonk, NY.

The primary outcome variable of interest was DSD. The independent variables of interest were BMI, smoking exposure as determined by cotinine levels, age, race/ethnicity, income, insurance status and educational achievement.

Chi square tests were used for comparison of proportions of subjects with DSD by various sociodemographic variables. Univariate analyses were initially conducted to determine which of these variables were significantly associated with DSD. All variables significantly associated with DSD were then included in the multiple logistic regression analyses to determine which variables were independently predictive of DSD while controlling for all the other confounders. A *p*-value of < 0.05 was as a test of significance in all cases.

## Results

### Socio-demographic characteristics of the study population

A total of 5,470 subjects met the study criteria. The average age of the population was 45 years with a range of 16–80+ years, while the mean BMI was 28.5 with a range of 13.40–82.10. Serum mean cotinine and vitamin D levels were respectively 41.2 ng/mL (range of 0.01–1700) and 25.5 ng/mL (range of 3.3–150). White subjects were 67% as compared to 33% non-white. The rest of the Table is self-explanatory (also see Table 1).

**Table 1:** Sociodemographic characteristics of the study population.

**Variable (weighted, *N =* 5,470)**	**Mean (95% CI)**	**Range**	**SD**
Age (yrs.)	45.3 (43.7–47.0)	16–80+	19.4
BMI	28.5 (28.0–29.0)	15.4–82.1	6.9
Cotinine Levels (ng/mL, *N =* 5,413)	41.2	0.01–1700.0	116.8
Vitamin D Levels mg/dL (*N =* 5,513)	25.5	3.3–150.0	11.3
**Race % (95% CI)**			
Mexican Americans	8.2 (5.1–12.8)		
Other Hispanic	6.6 (4.0–10.5)		
White Americans	66.6 (57.6–74.0)		
African Americans	11.2 (7.2–17.2)		
Other	7.7 (5.6–10.3)		
**Gender % (95% CI)**			
Males	48.4 (47.0–49.8)		
Females	51.6 (50.2–53.0)		
Income ($)	78,000 (70,000–86,000)		
Insurance (Any)	80.5% (77.3–83.3)		
Education > 4^th^ Grade	62.4 % (56.0–68.3)		

### Prevalence of DSD by socio-demographic characteristics

**Figure 2:**
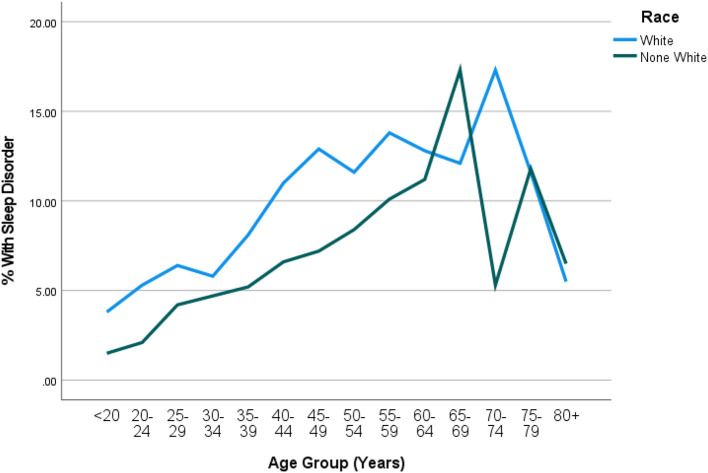
Weighted prevalence of DSD by race and age-group. DSD rose steadily by age group and was consistently higher among white than none-white in all age groups below 60 years (*P* < 0.001). The highest peak of DSD occurred earlier among none-white in the 66–69 year age group (17%) but among white the peak occurred later in the 70–74 year age group (17%).

Out of 5,740 subjects who met the study criteria, the prevalence of subjects who consulted their doctor for sleep problems was 26% (95% CI = 23.4–28.6%). However, only 8.6% (95% CI = 7.5 = 10.0) were diagnosed with a sleep disorder by a physician. In the univariate analyses eight sociodemographic characteristics were explored to determine those associated with DSD. Older subjects were more likely to suffer from DSD than their younger counterparts. DSD gradually increased with age until after 60 years followed by significant drop especially among men (also see Figure 2). BMI was highly predictive of DSD with those in obese range more likely to suffer from DSD as compared to the non-obese (*P* < 0.001). Generally, the higher the BMI, the higher the prevalence of DSD. Thus, subjects in the class II or class III obesity group were more than 20 times more likely to have DSD than those with a BMI in the non-overweight range exhibiting a significant dose-response relationship. When tobacco smoke exposure was categorized by no exposure (cotinine < 0.05, referent group), SHS exposure (cotinine 0.05–10 ng/mL), and active smoking (cotinine >=10 ng/mL), DSD prevalence increased respectively to 7.7 and 11.7% (*P* < 0.001). Having any type of insurance (private or Medicaid) was predictive of DSD (*P* = 0.016). Factors not associated with DSD were gender, vitamin D, and income (also see Table 2).

**Table 2:** Prevalence of doctor-diagnosed sleep disorder (DSD) by socio-demographic characteristics among subjects 16–80+ years in the United States.

**Variable (*N =* 5,470)**	**Weighted % of DSD (95%CI)**	***P*–value**
**Age (Yrs.)**		
< 20	3.0 (1.7–5.4)	
20–35	4.9 (3.4–7.0)	
36–55	9.6 (6.9–13.0)	
56+	11.5 (9.8–13.4)	< 0.001
**Gender**		
Male	9.4 (7.6–11.5)	
Female	8.0 (6.4–9.9)	0.294
**Race**		
White	9.9 (8.4–11.6)	
Non-white	6.2 (5.1–7.6)	< 0.001
**BMI**		
Not Obese and not Overweight (< 25)	4.87 (3.3–6.6)	4.87 (3.3–6.6)
Overweight (25–29.99)	6.9 (5.1–9.3)	
Obesity Class I (30–34.99)	9.1 (6.3–13.0)	
Obesity: Class II (35–39.99)	21.3 (16.3–27.4)	
Obesity: Class III (40+)	20.9 (15.6–27.5)	< 0.001
**Smoking status (Cotinine in ng/mL)**		
Unexposed (< 0.05)	7.8 (6.5–9.5)	
Exposed to SHS (0.05–10)	7.4 (6.3–10.3)	
Active smoking (10+)	11.7 (8.9–15.1)	0.024
**Vitamin D levels (ng/mL) All**		
Deficient (< 20)	9.5 (7.6–11.8)	
Insufficient (20–29.99)	7.2 (5.6–9.1)	
Sufficient (30+)	9.4 (7.4–12.0)	0.152
**Income ($)**		
Average (< 78000)	8.7 (7.0–10.7)	
Average + (>= 7800)	9.1 (8.1–10.2)	0.663
**Insurance**		
Yes	9.2 (8.1–10.5)	
No	6.3 (4.4–8.9)	0.016

Significance P: based on the Rao-Scott adjusted chi-square statistic. Significance is based on the adjusted F and its degrees of freedom).

### Prevalence of DSD by age, gender, and race/ethnicity

**Figure 3:**
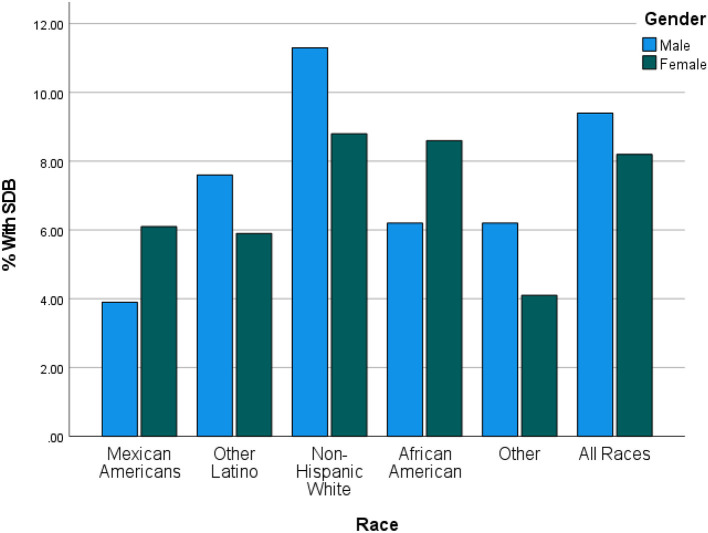
Prevalence of DSD by race and gender in the United States. Generally DSD was slightly higher in males than females except in Mexican Americans and in African Americans but the sex difference in each of the racial groups was not statistically significant (*P* > 0.05).

**Figure 4:**
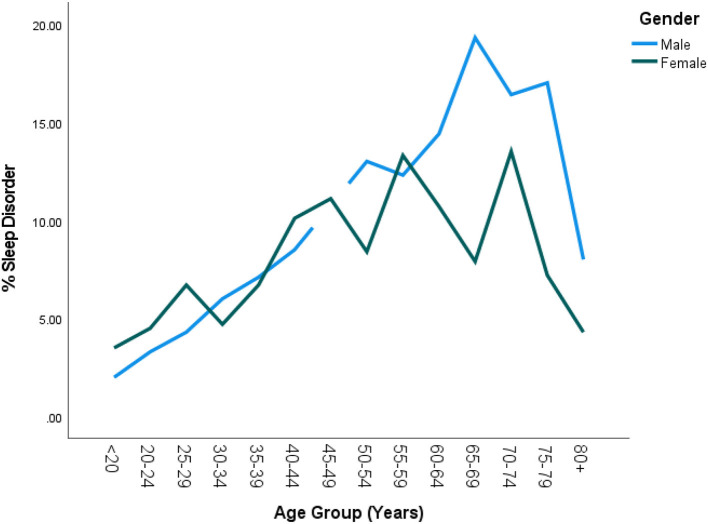
Prevalence of DSD by age group and gender in the United States. There was generally no significant sex difference in DSD until the age of 60 after which males had significantly higher rates of DSD than females (*P* < 0.05). Furthermore, DSD prevalence among females began to drop by 60 years as compared to the males whose rates only started dropping by 65 years +.

DSD was higher among white Americans (9.9%; 95% CI = 8.4–11.6%) than their non-white counterparts (6.2%; 95% CI = 5.1–7.6%; also see Table 2). This was true in all age groups only up to 60 years (*P* = 0.015). Table 3 shows the details of the ethnic distribution of DSD. Also, the peak prevalence of DSD for both White and non-White was exactly 17.3% but occurred earlier for non-White (65–69 years) than for White subjects (70–74 years; also see Figure 2). In each of the racial groups, males were slightly more likely to have DSD than their female counterparts except among Black Americans and Mexican Americans although differences were not significant in any of the racial subgroups (*P* > 0.05; also see Figure 3). However, among subjects older than 60 years, males were significantly more likely to have DSD than their female counterparts (*P* < 0.05; also see Figure 4).

**Table 3:** Prevalence of DSD among US adults by race/ethnicity in the USA.

***N** **=*** **5,470**	
**Weighted prevalence of subjects with trouble sleeping** = **26% (95% CI: 23.4–28.6%)**	
**Weighted prevalence of DSD** = **8.6% (95% CI: 7.5–10.0%)**	
**Race/Ethnicity**	**Weighted prevalence of DSD by race, % (95% CI)** ^*^
Mexico Americans	5.0 (2.9–8.5)
Other Hispanic	6.6 (4.8–8.9)
Whites (Non-Hispanic)	9.9 (8.4–11.6)
African American	7.7 (6.2–9.5)
Other	5.2 (3.2–8.5)

^*^P = 0.003 (Based on the Rao-Scott adjusted chi-square statistic. Significance is based on the adjusted F and its degrees of freedom). The highest prevalence rate of DSD was noted in Non-Hispanic White Americans (10%) followed by African Americans (8%), while the lowest rates was among Mexican Americans (5%).

### Multiple regression analyses of factors associated with DSD

In a multiple regression analysis, all 5 factors predictive of DSD were included in the analysis in order to control for confounders. Subjects in the Class II (BMI of 35–40) or III (BMI = 40+) obesity groups were 5 times more likely to suffer from DSD than those with BMI < 25. Overweight (BMI of 25–30) was not predictive of DSD. Active smoking remained significant in the multivariate analysis (but passive smoking was no longer predictive of DSD even though it was predictive in univariate analysis. Insurance was no longer predictive of DSD (also see Table 4).

**Table 4:** Multiple logistic regression of factors associated with DSD in the US 16- 80+ years (final model).

**Variable**	**OR (95% CI)**
**BMI**	
< 25 (Non-obese, Referent)	1
25–29.99 (Overweight)	1.2 (074–2.03)
30–34.99 (Class I Obesity)	1.80 (1.11–2.90)
35–39.99 (Class II Obesity)	5.40 (3.23–9.01)
40+ (Class III Obesity)	5.50 (3.17–9.57)
**Age in years**	
16–19 (Referent)	1
20–35	1.76 (0.83–3.76)
36–55	3.19 (1.67–6.11)
56+	4.11 (2.18–7.74)
**Smoking exposure status (Cotinine levels, ng/ml)**	
Not exposed (< 0.05, Referent)	1
Exposed (0.05–10)	0.93 (0.57–1.52)
Active smoking (10+)	1.92 (1.38–2.69)
Race (white vs. non-white)	1.46 (1.11–1.92)
Insurance, any (yes vs. none)	1.40 (0.92–2.12)

## Discussion

In this population-based national representative sample of adults in the United States, our study clearly demonstrates the following 4 important findings: (1) the prevalence of DSD was similar to previous studies; (2) the study confirms the association of traditional risk factors with DSD: BMI, age, race and/or ethnicity; (3) when smoking exposure was objectively defined by cotinine levels active smoking was clearly demonstrated as an important risk factor of DSD but SHS exposure was not, even after controlling for all the other well-known confounders such as BMI, age, gender, and race/ethnicity; and (4) Vitamin D deficiency was not a significant predictor of DSD among the general population in the United States.

The prevalence of physician-diagnosed DSD in our study was close to 9% which is similar to two earlier studies in the US using polysomnography (Young et al., 1993, 2002). It is not clear if polysomnography was used in all cases in this dataset but the prevalence of only 9% (out of the 26% who had sleep problems) suggests that sleep disorder diagnosis by the physicians in this study was very stringent as would be expected if polysomnography and other tests was utilized. It would seem that prevalence of sleep disorder is much higher in some European studies. For instance, in one study conducted in Spain, 19% of men and 14% of women had OSA (Young et al., 2002). In another study in Switzerland, 23.4% of women and 48.9% of men were diagnosed with a sleep disorder (Heinzer et al., 2015). Differences between these studies are probably, due to differences in definitions and populations studied. For instance, in the study by Bixler et al. (2001). when OSA was defined as AHI of 15+, the prevalence of OSA was much lower than the Spanish study which defined OSA as AHI of 10+ (Young et al., 2002). Another reason for the lower prevalence in our study was the fact that it included younger subjects and it is well known that older age groups are much more likely to develop a DSD (Young et al., 1993; Ralls and Grigg-Damberger, 2012).

The finding that individuals with any form of insurance were likely to have DSD was counter-intuitive. It is unclear why individuals who have insurance would have a higher prevalence of DSD as compared to individuals without insurance. It could be assumed that individuals who have any form of insurance as compared to their uninsured counterparts have greater access to healthcare and have a significantly increased likelihood of being diagnosed with a sleep disorder. Other studies looking more precisely at access to healthcare as a variable with regard to risk of DSD might clarify this counter-intuitive finding. Indeed after controlling for various confounders, insurance was no longer a significant predictor of DSD.

Our study clearly shows that active tobacco smokers were twice more likely to develop DSD than their non-smoking counterparts even after controlling for all the other selected confounding sociodemographic variables. However, SHS exposure as defined by cotinine levels was not associated with DSD in our study. Interestingly, passive has been shown to also be predictive of DSD in some other studies (Nakata et al., 2008; Sabanayagam and Shankar, 2011). In these studies SHS exposure was defined by history alone without objective validation with cotinine levels. Therefore, it is possible that those who were heavily exposed to SHS exposure could have had cotinine levels in the smoking range.

How tobacco smoking may cause DSD is still a matter of some speculation. Tobacco smoke contains about 7000 toxins and nicotine is one of the most important intoxicants (Krishnan et al., 2014). Nicotine has been shown to cause increased inflammation of the upper airways such as increased swelling of the mucosal membranes of the nose and throat leading to decreased air flow in these airways resulting in sleep disruption (CDC, 2017). Nicotine may act by suppressing the cardiorespiratory center located in the midbrain thus decreasing the sleep arousal mechanism during hypoxic stress when sleeping (Berry and Gleeson, 1997). It has been further shown that nicotine suppresses the arousal responses to hypoxic stress during sleep *via* the beta-2 nicotinic acetylcholine receptor subunits. In mutant mouse lacking these beta-2 acetylcholine receptor subunits, nicotine had no effect on sleep disruption (Cohen et al., 2019).

That observation that BMI was the most significant predictor of DSD was not surprising as almost all studies supported this finding (Davies and Stradling, 1990; Shinohara et al., 1997; Young et al., 2005; Shneerson, 2012). This study confirms the finding that BMI was the most significant predictor of DSD and also demonstrated a dose-response relationship confirming most previous studies (Arias et al., 2005; Leinum CJ, 2009). The mechanisms by which obesity causes sleep disorder has also been discussed by various authors (Series et al., 1995; Schwartz et al., 2008; Ryan et al., 2014).

In our study, however, DSD was more prevalent among White Americans than other races even after controlling for the various confounders. This was rather surprising as most small epidemiologic studies show that obstructive sleep apnea is more common in Black Americans and other minority populations than in White Americans (Wolk et al., 2003; Ruiter et al., 2010; Chen et al., 2015). We believe that further studies are needed to determine the true ethnic differences of DSD in the US. One reason for this discrepancy could be the lack of information on how DSD was diagnosed by the various physicians in this study. The standard is to perform polysomnography for accuracy but it is possible that some of the diagnoses were made clinically without objective validation by polysomnography, possibly based on symptoms alone. Another important reason could be that studies delimited to certain age groups could demonstrate varying ethnic differences. For instance, using in-home monitoring, the study by Ancoli-Israel et al. among 65+ years community dwellers found that Black Americans were 2.5 times more likely to have OSA than White Americans (Redline et al., 1997). Would in-home monitoring for population-based study have produced the same results? Indeed, Young et al. demonstrated that SDB was similar between Black and White Americans when in-home monitoring was conducted controlling for the various important known confounders (Young et al., 1993). Ethnic differences of SDB need to be further explored.

The association of vitamin D and DSD has often produced contradictory results. In our study vitamin D was not predictive of DSD. In some small studies vitamin D has been reported as being associated with SD (Mete et al., 2013; McCarty et al., 2014; Kerley et al., 2016). In one such study, vitamin D (25(OH)D) deficiency was predictive of OSA among a Caucasian population of an urban population in Dublin, Ireland (Kerley et al., 2016). It is therefore possible that this difference was due to the differences in the less diverse Irish study as compared to our study. More studies are needed to determine the real impact of Vitamin D on DSD.

This study has at least 2 limitations. First, it was a cross-sectional survey so any associations cannot be deemed causal. Second, as previously stated above, physician-diagnosed DSD is not well defined as it is not clear if objective validation of DSD was made by polysomnography in all cases. Additionally, the assumption that individuals who answered “yes” to the very non-specific question, “Ever told by doctor to have sleep disorder?”, may be significantly flawed in that the individual might have been told by a doctor that they have one or more of a multitude of sleep disorders other than those attributable to the subset term SDB or OSA given that there are more than 81 diagnosable sleep disorders per the latest version of the International Classification of Sleep Disorders – Third Edition (ICSD-3) (American Academy of Sleep Medicine., 2014).

However because the prevalence in this population was only 8.6%, which is in agreement with other studies that were conducted using polysomnography and other diagnostic tests, it is reasonable to conclude that polysomnography was likely used for the most part in making the diagnoses in this large epidemiologic study (Young et al., 1993, 2002). Furthermore, the strength of the study lies in the circumstance that it was a population-based study representative of the United States.

## Conclusion and recommendations

The prevalence of physician-diagnosed DSD was about 9%. The present study demonstrates that active smoking, but not passive smoking, was predictive of DSD among a representative sample of the adult US population even after controlling for selected sociodemographic confounders. Vitamin D was not predictive of DSD as previously determine in some small studies. We recommend that more studies be conducted to determine the true impact of Vitamin D levels on DSD in the United States.

## Data availability statement

The original contributions presented in the study are included in the article/supplementary material, further inquiries can be directed to the corresponding author.

## Ethics statement

The studies involving human participants were reviewed and approved by the Virginia Commonwealth University School of Medicine, Richmond, Virginia. The patients/participants provided their written informed consent to participate in this study.

## Author contributions

PK-N: conceived the project, data curation, draft manuscript write up, and data analysis. ST: data analysis, draft write up, and editing of final manuscript. BT: draft manuscript writing, data editing, and final editing. All authors contributed to the article and approved the submitted version.
